# Communication pathways from the emergency department to community mental health services: A systematic review

**DOI:** 10.1111/inm.13024

**Published:** 2022-05-22

**Authors:** Heather McIntyre, Verity Reeves, Mark Loughhead, Laura Hayes, Nicholas Procter

**Affiliations:** ^1^ University of South Australia Adelaide South Australia Australia; ^2^ MIND Australia Heidelberg Victoria Australia

**Keywords:** communication pathways, continuity of patient care, emergency service, hospital, lived experience, Psychosocial disability

## Abstract

This systematic review synthesizes existing peer reviewed evidence reporting on evaluated strategies used for enhancing communication pathways for continuity of care between the emergency department and mental health community supports. Following the PRISMA guidelines and the PICO framework, this review was conducted between January and July 2021. Included articles needed to evaluate communication pathway interventions for continuity of care between the emergency department and mental health community services which support service users with mental health and/or suicidal crisis. The seven included studies identified three support coordination interventions, two motivational interviewing interventions, an electronic record enhanced strategy and results from a phone follow‐up study. This review demonstrates that support coordination, motivational interviewing, education, or an enhanced electronic record strategy can improve continuity of care, and in some cases, reduce the need for people to re‐present to ED when they are experiencing mental health concerns or suicidal crisis. Results of this review reveal that a multipronged approach of communication pathways for continuity of care would enable more effective connections with mental health community supports and enable better outcomes for people requiring services.

## BACKGROUND

People with a psychosocial disability presenting to the emergency department (ED) can have many challenges co‐occurring that have led to an acute crisis. These can range from health comorbidity, housing instability (Kaplan & McGrath 2018; Royal Australian & New Zealand College of Psychiatrist 2015), relationship breakdown, substance use (Hielscher *et al*. 2019), disconnection with support networks or difficulties in navigating access across multiple services (Duggan *et al*. 2020). In addition, they could have presented repeatedly to the ED over several days (Office of the Auditor General 2019) without gaining the assistance required due to inadequate or rationed care (Morris 2021). Furthermore, they may be discharged without follow‐up care arranged (Australasian College for Emergency Medicine 2018; Dayman 2020; Magarey 2019) and be at greater risk of dying by suicide after discharge (Chung *et al*. 2019; Forte *et al*. 2019). This concerning context highlights the need to improve pathways of communication and continuity of care between these providers and the service users seeking assistance in crisis (Wierdsma *et al*. 2009).

Continuity of care is a multi‐layered concept that incorporates communication pathways to enable and promote person‐centred care (Pereira *et al*. 2016). Designed to improve the quality of healthcare and service outcomes, continuity of care promotes enhanced connections across the relational and administrative parts of organizational systems, enabling health care to be offered in a timely and personalized manner as required by the individual (Wierdsma *et al*. 2009).

Since the 1990s, mental health care has been mainstreamed into the general health care sector. Driven by consumer rights movements, and other forces for deinstitutionalisation, this integration of services has seen constant challenges in providing high levels of care and connection between EDs and specialist mental health supports. EDs in Australia are constantly stretched beyond limits, with mental health presentations, especially after business hours when community supports may not be available (Duggan *et al*. 2020). Therefore, failures of service provision have occurred due to services reaching capacity and beyond (Morris 2021). Although more likely to be triaged by ED staff as requiring ‘Urgent Care’, people with a mental health presentation have longer waiting times, treatment times (11.5‐h average compared to within 7 h for medical presentations) and more likely to leave at their own risk (Australian College of Emergency Medicine 2018; Duggan *et al*. 2020; Mental Health Commission 2020). One person's experience can be seen here:

I have sat in distress in ED on multiple occasions. Between the bright lights, yelling, police, pain and chaos of the surroundings – and my distress – I begin pacing, humming, tapping…just to try and block it out. Due to the long wait, I am either chemically restrained because of my distress, or repeatedly pressured to ‘calm down’, which funnily enough does not work. By the time I have waited [for] eight to 13 hours to speak to someone from mental health, I am silenced by the medication, by privacy concerns, and by the increase in my distress…I was discharged from ED. It was 5 am, I had been awake for three days and felt unworthy of help. Sitting in my car I began self‐harming and phoned crisis support, who referred me [back] to ED. I tried multiple hotlines and kept getting the same advice. I felt so hopeless, overly worthless and almost supported in my decision to end my life. Retrospectively I can [now] explain that feeling as having my pain invalidated (Consumer) (Australian College of Emergency Medicine 2018, p. 3).

The quotation above reveals the experience of a service user^1^ in an underfunded, overstretched health system in failure, resulting in an exacerbation of symptoms and distress, culminating in an acute suicidal crisis at point of care (Australian College of Emergency Medicine 2018).

Emergency departments are often considered the ‘canary in the coal mine’ in identifying failures in the health system…mental health system fails individuals, families and health services, and that the strain on emergency departments and, more importantly, patients and families, is unsustainable (Duggan *et al*. 2020, p. iii).

ED clinical staff are not necessarily highly trained in mental health care or trauma‐informed practice (Fischer *et al*. 2019; Molloy *et al*. 2020). Consequently, restraint and seclusion are excessively used on adults and children when in a mental health or suicidal crisis in the ED (Dayman 2020; Magarey 2019). This concerning phenomenon is explained here:

Our processes are focused more on containment than recovery because of two main reasons: one, there are sanctions against allowing patients to 'escape' but not against traumatising the patient; and two, EDs cannot have perimeter security (Duggan *et al*. 2020, p. 17).

23 000 people present to an ED within Australia each day; 3.6 per cent are people with a mental health presentation (AIHW 2018, AIHW 2019a, 2019b; McIntyre *et al*. 2021). For some consumers, long waiting times and slow responses lead them to leaving EDs without full assessment or care plans. When people leave, it is recorded in their notes that they ‘left at own risk’ (ACEM 2018) (7000 people left EDs prior to receiving treatment in 2016/17). While EDs record statistics on people who present with mental health concerns who ‘leave at own risk’ (ACEM 2018), statistics are not readily accessible which report numbers of people being discharged without any follow‐up care organized.

Carers who attend the ED with the person that is presenting come with an expectation that effective and compassionate mental health care will be provided (Acres *et al*. 2021) including after care (follow‐up). The *National Standards for Mental Health Services* clearly upholds the human rights of people who are entering the mental health system, affirming that they are partners of their own healthcare which should occur in a safe environment and in a timely manner (Australian Government 2010). Therefore, it is vital that research focused on communication pathways and continuity of care involves and values people with lived experience (Daya *et al*. 2019; NHMRCCHFA  2016). This is essential to address inequities in human rights for people in distress and to evaluate quality of care pathways.

## AIMS

The aim of this review is to synthesize all existing international literature regarding communication pathways for continuity of care from the ED to community mental health services, outpatients and/or general practitioners for people presenting to the ED with mental health concerns or suicidal crisis. This review informs ED clinicians, community mental health teams, consumer advocates and disability support providers of results of researched communication pathways to enhance continuity of care to support engagement for people with lived experience between ED and community care settings.

Research questions:

What evaluated strategies are used for enhancing communication pathways for continuity of care between the emergency department and mental health community support services for consumers experiencing mental health crisis?

How have lived experience perspectives on continuity of care between the emergency department and mental health community support services been analysed and recorded in these included studies?

The systematic review questions seek to understand elements that assist good communication practices between ED clinicians and people working in community mental health services, outpatients, or general practitioners. The review collates and synthesizes the literature which reports results from any clinical trial or qualitative research, including studies that elicit voice of people with lived experience. Documenting the lived experience voice provides a greater understanding of lifeworld perspectives of service users and carers (Johnston *et al*. 2019) and inform clinicians of preferred processes in this context (Banfield *et al*. 2018; Robotham *et al*. 2016). The lived experience perspective brings deep awareness of, and understanding about, events and experiences within the ED and connection with follow‐up care to inform clinicians of service gaps and how best to meet their needs (Suomi *et al*. 2020). Such insights are a key contribution as to the conceptualisation of this research, as well as the analysis of the data, and echoes the findings of earlier results of research priorities for people with lived experience (Banfield *et al*. 2018; Robotham *et al*. 2016). The research team for this review includes a lived experience researcher and a lived experienced carer.

## METHOD

As this review is part of a larger study, a consultation with people with lived experience and sector leaders occurred. Utilizing the personal experiences within the ED in a crisis has enabled the research team to reflect on the research questions and narrow the research focus.

### Search strategy

The search strategy was developed by the research team along with guidance from an academic librarian and was conducted in January 2021. Using the PICO framework (Population, Intervention, Comparison, Outcome) to design the search strategy, it was conducted in the following databases: Ovid Embase, Ovid Emcare, Ovid Joanna Briggs Institute, Ovid Medline, Ovid Nursing, Ovid PsycInfo, Scopus and Web of Science. Variations of MeSH and keywords were used: mental health, emergency department, continuity of care, community mental health services and communication pathway. Databases hosted by Ovid enabled limiting search terms to MeSH and keywords in titles abstracts and keywords. Hand searching and consulting reference lists of all included articles were conducted to seek further potential results. All articles were imported into EndNote and duplicates were removed.

### Eligibility criteria

**Fig. 1:**
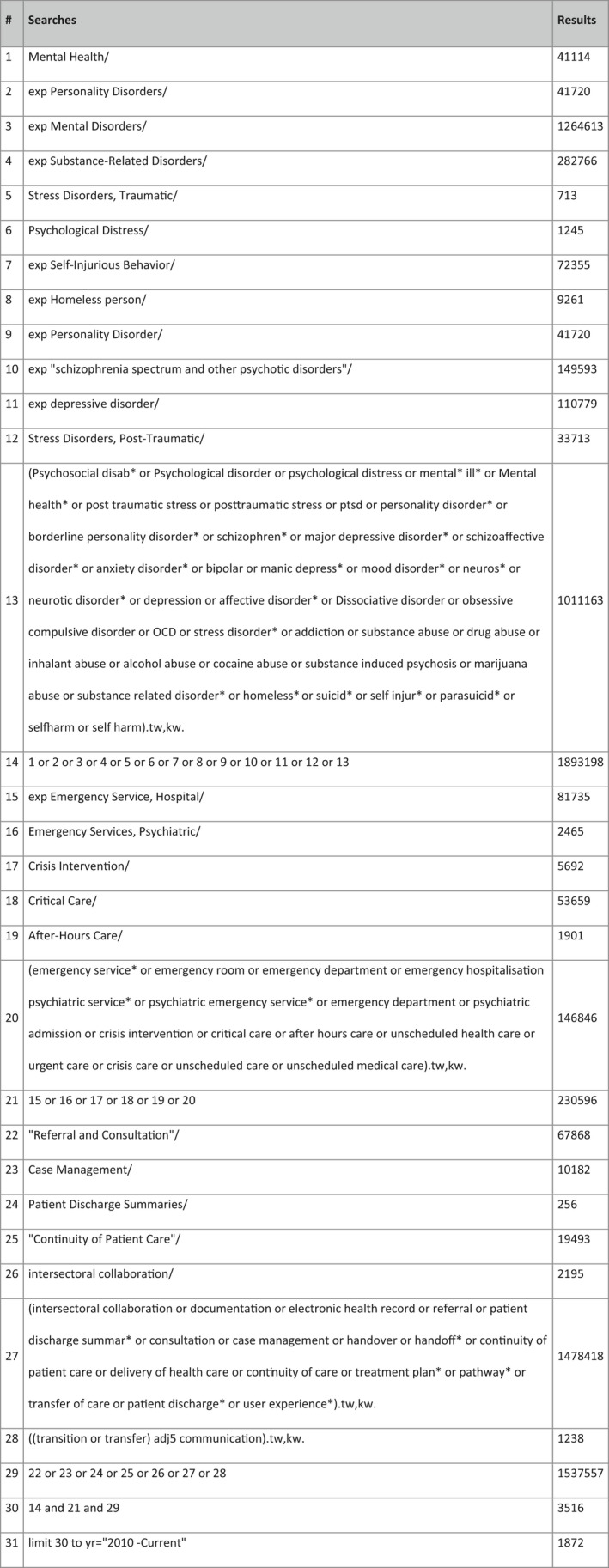
Search Strategy. Ovid MEDLINE(R) ALL 1946 to January 15, 2021.

Qualitative and quantitative studies that were eligible included people with mental health or suicidal crisis presenting to an ED, where the focus of the intervention was a communication pathway to enable continuity of care between the ED and community mental health services, outpatients and/or general practitioners (see Fig. 1). The research team limited the search to articles published since 2010 to capture more recent interventions around presentations to EDs. All languages were included, plus participants of all ages as paediatric mental health is a growing concern^2^.

Studies were excluded if they were not published in a peer reviewed journal, editorials, conference abstracts, case studies, commentaries, reviews, descriptive and opinion pieces. Studies were also excluded if communication was between an ED and an inpatient facility as this review specifically focussed on service users being discharged from the ED (without having any inpatient care). Our focus was strategically around pathways and procedures at transfer of care from the ED to community mental health care, outpatients, or general practitioners (i.e. situations where there was a ‘step down’ from ED, rather than a ‘step up’).

### Study selection

Screening and study selection followed a three‐stage process: (a) HM searched the databases (9224 results including hand searching). Duplicates were removed (4176) and the remaining articles were then imported from EndNote into Covidence (Veritas Health Innovation 2018) to allow for blind review (5048 results). (b) HM and VR screened all remaining article titles and abstracts and excluded results that were not matching the inclusion criteria (4948 results); (c) The full text of articles (100 results) that were retained after the title and abstract screen were then read and reviewed by HM and VR. Any discrepancies between HM and VR around inclusion/exclusion were discussed to achieve 100% consensus. Results included 10 research papers reporting on seven studies to be included in the review.

### Data extraction

**Fig. 2:**
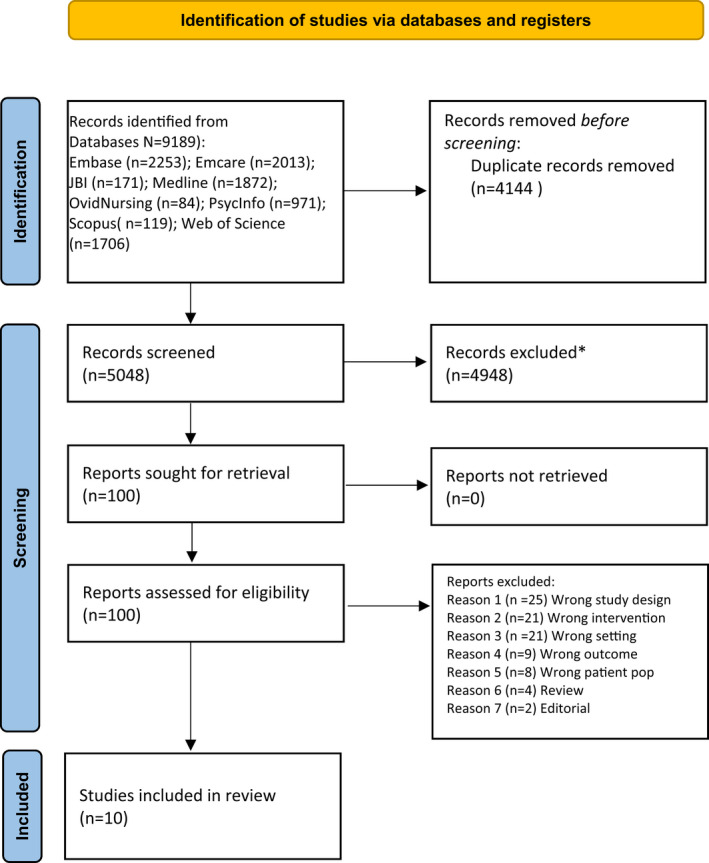
PRISMA Flow chart (Page *et al*. 2021).

Data were extracted following PRISMA guidelines (see Fig. 2) in these domains: study design; country; sample size; age; gender; follow‐up; intervention/measures and outcomes (Page *et al*. 2021). This was conducted by HM and cross‐checked by VR.

### Risk of bias

Quality appraisal assessments for methodological quality were conducted and due to the variations of study designs, three risk of bias critical appraisal checklists were used (Joanna Briggs Institute). The JBI *Checklist for randomized controlled trials* (Tufanaru *et al*. 2020), *Checklist for quasi‐experimental studies* (Tufanaru *et al*. 2020) and the *Checklist for qualitative studies* (Lockwood *et al*. 2015) were used. Each of these tools have a series of questions (13, 9 and 10, respectively) with one point being given for a ‘Yes’ response. Other responses are ‘No’, ‘Unclear’ or ‘Not Applicable’. The research team agreed on the following definitions: 85–100% = high quality; 70–84% = moderate to high quality; 55–69% = moderate quality (Munn *et al*. 2020; Porritt *et al*. 2014) and if a study scored 50% or lower, it would be excluded from the results. This process was completed by HM and VR cross‐checked 20% of the papers for inter‐rater confidence and reliability (Richardson *et al*. 2021) and can be seen in Table 1.

**Table 1:** Critical appraisal

	Citation	1	2	3	4	5	6	7	8	9	10	11	12	13	%	Score
QN1	Adams & Nielson (2012)	*	*	*				*	*						5/9	55%
QN2	Esposito *et al*. (2020)	*	*	*	*	*	*	*	*						8/9	88%
QN3	Griswold *et al*. (2010)	*	*	*	*	*		*	*	*	*	*	*	*	12/13	92%
QN4	Grupp‐Phelan *et al*. (2019)	*	*	*		*	*	*	*	*		*	*	*	12/13	92%
QN5	Grupp‐Phelan *et al*. (2012)	*	*	*			*	*	*	*	*	*	*	*	11/13	84%
QN6	Hopper *et al*. (2011)	*	*	*			*	*	*						6/9	66%
QN7	Stergiopoulos *et al*. (2017)	*		*	*			*	*	*	*	*	*	*	10/13	77%
QL1	Kahan *et al*. (2016a)	*	*	*	*	*			*	*	*				8/10	80%
QL2	Kahan *et al*. (2016b)	*	*	*	*	*			*	*	*				8/10	80%
QL3	Poremski *et al*. (2016)	*	*	*	*	*			*	*	*				8/10	80%

### Synthesis of results

Due to the heterogeneous nature of the interventions, methodologies and outcomes, the included studies are presented in a narrative summary. This review also reports if any study included lived experience participants or lived experience co‐researchers or advisors.

### Study characteristics

Characteristics of the 10 included articles (representing seven studies) are summarized in Table 2 and the data extraction can be viewed in Table 3. All studies were situated in the ED and reported on interventions that were categorized as communication pathways for continuity of care at transfer of care to community mental health services, outpatients or to general practitioners. The results of this review consider three support coordination (SC) interventions: an improvement plan (Adams & Nielson 2012), Care navigators (Griswold *et al*. 2010), and CATCH‐ED (Stergiopoulos *et al*. 2017); two motivational interviewing interventions with SC: STATE‐ED (Grupp‐Phelan *et al*. 2019) and TeenScreen (Grupp‐Phelan *et al*. 2012); an electronic record enhanced strategy (Esposito *et al*. 2020) and results from a phone follow‐up intervention (Hopper *et al*. 2011). One study provided qualitative data following an RCT (Stergiopoulos *et al*. 2017) (Kahan *et al*. 2016a, 2016b; Poremski *et al*. 2016). Participant numbers ranged from 24 to 783 (total 1426 participants) with one study (a medical record review) not stating numbers (Adams & Nielson 2012) (Mean: 178; Median: 107).

**Table 2:** Study characteristics and interventions

#	Country	Study type	# Site	I/view	FG	P#	Participants	Repeat Presenters	ED connection	Gender reported	Results	LExV	LER	PS	Age/Screen tool	IntVN type/name
QN1	USA	Qi	1	No	No	NS	MH	Yes	OP	NS	Pre intervention re‐visits to ED: = 6.51% Post intervention re‐visits to ED: = 4.3%	No	No	No	NS	SC and slowing down throughput
QN2	USA	Qi	1	No	Yes	*N* = 783	MMC/SD or Su	Yes	PCP	F: *n* = 96 (79.3%)	Pre intervention re‐visits to ED: = 4% Post intervention re‐visits to ED: = 0.5%	No	No	No	14–19 BHS‐ED	Electronic record enhanced strategy ERES
QN3	USA	RCT	1	No	No	*N* = 101/ 151	MH	No	PCP	UC F: 31 Intervention F: 36	Access to care post ED: UC 37.6% Intervention: 62.4%	No	No	Yes	19–80	Care navigator SC
QN4	USA	RCT	1	No	No	*N* = 159	MMC/SD or Su	No	CMHS	F: *n* = 126 (79.2%);	MH treatment initiation post ED: UC = 34.9% Intervention = 50.9% Treatment attendance post ED, 1 appointment: UC = 3.6% Intervention 9.7% Treatment attendance post ED, >2 appointments: UC = 12.7% Intervention = 16.1%	Carer/ Service user	No	No	12–17 ASQ	STATE‐ED MI & SC
QN5	USA	RCT	2	No	No	*N* = 24	MMC/SD or Su	No	CMHS	Standard F: 10 (76.9%) TeenScreen ED: F: 9 (81.2%)	Treatment attendance post ED: UC = 15.4% Intervention = 63.6%	Parent/Service user	No	No	12–17 CSS	TeenScreen‐ED MI & SC
QN6	Australia	Qi	1	No	No	*N* = 113	SI or SA	No	CMHS and PCP and OP	F: 77%	Treatment attendance post ED: UC = 83.1% Intervention = 85.8%	No	No	No	Median 14	Phone follow‐up PH
QN7	Canada	RCT	6	No	No	*N* = 186	MH	Yes	CMHS and PCP	CATCH‐ED: F: 44 (53%) UC: F: 37 (44.6%)	Frequency ED visits over 12 months: ED revisits reduced by 14% (NS) in intervention condition	No	No	Yes	18+	CATCH‐ED SC
QL1	Canada	Qual	6	Yes	Yes	*N* = 47	SI or SA	Yes	CMHS and PCP	NS	See data extraction	No	No	Yes	NS	CATCH‐ED SC
QL2	Canada	Qual	6	Yes	Yes	*N* = 33	MH	Yes	CMHS and PCP	NS	See data extraction	Service user	Yes	Yes	18+	CATCH‐ED SC
QL3	Canada	Qual	6	Yes	Yes	*N* = 33	MH	Yes	CMHS and PCP	NS	See data extraction	Service user	No	Yes	18+	CATCH‐ED SC

Abbreviations: Adol, adolescent; ASQ, Ask suicide screenings; BHS‐ED, Behavioural Health Screen; CMHS, community mental health services; CSS, Columbia suicide scale; ERES, electronic record enhanced strategy; FG, focus group; I/view, interview; IntVN, intervention; LER, lived experience researcher; LExV, lived experienced voice; MH, mental health; MI & SC, motivational interviewing and support coordination; MI, motivational interviewing; MMC, mild medical complaint; OP, outpatients; P#, population; PCP, primary care physician (GP); PH, phone follow‐up; PS, peer support; Qi, quasi‐experimental; Qual, qualitative; RCT, randomized controlled trial; SA, suicide attempt; SC, support coordination; SD, seriously depressed; SI, suicidal ideation; Su, suicidal; Sur, surveys.

**Table 3:** Data extraction

Citation	Study design	Country	Sample size	Age mean	Female %	Follow‐up	Intervention	Outcome
Adams & Nielson (2012)	Quasi‐experimental	USA	NS Record review	NS	NS	Revisits within a month	Improvement plan – support coordination	Mean revisits to ED 6.51% in 2010 to 4.3% in 2011
Esposito *et al*. (2020)	Quasi‐experimental	USA	*n* = 783 Record review	14–19 years	F: *n* = 96 (79.3%)	Revisits within a month	Electronic alert flagging recommendation to communicate with PCP† Communication to PCP PCP able to view results of the BHS‐ED	Discharged from the ED with mental health follow‐up recommended 0.5% returned to ED within 4 weeks compared with 4% when no PCP communication occurred
Griswold *et al*. (2010)	RCT	USA	*n* = 151	19–80 years (average 37.5) SD 11.6.	F: 44%	1 year	Care Navigator ‐ Support coordination (appointments, phone calls, referrals, home visits) Or usual care (psychiatric assessment therapeutic approaches, linkages to community Mental health services, and if requested referral to primary medical care	*n* = 101/151 (66.8%) achieved linkage to primary care 1‐year follow‐up Intervention: 63 (62.40%) UC: 38 (37.6%)
Grupp‐Phelan *et al*. (2019)	RCT	USA	*n* = 15	12–17 years (SD 1.5); Mean age 15	F: *n* = 126 (79.2%)	2 months	STAT‐ED intervention (motivational interviewing + follow‐up phone calls) Or UC (referral to psychiatric services)	Mental health treatment initiation: STAT‐ED: 29 (50.9%) EUC: 22 (34.9%) 1 appointment STAT‐ED: 6 (9.7%) EUC: 2 (3.6%) >2 appointments STAT‐ED: 10 (16.1%) EUC: 7 (12.7%)
Grupp‐Phelan *et al*. (2012)	RCT	USA	*n* = 24	Age 12–17: TeenScreen‐ED intervention – 13.3 (SD 1.7) Standard referral – 14.5 (SD 1.7)	Standard F: 10 (76.9%) TeenScreen ED F: 9 (81.2%)	2 months	TeenScreen‐ED (motivational interviewing) Or Standard referral (referral to psychiatric services)	TeenScreen‐ED: 63.6% attend mental health follow‐up appointment Standard referral: 15.4% attend mental follow‐up appointment (Fisher exact test, *P* = 0.03)
Hopper *et al*. (2011)	Quasi‐experimental	Australia	*n* = 113 Self‐harm 61%	Median age 14	F: 77%	3–7 days after discharge	Telephone follow‐up referral	*n* = 94/113 (83.1%) attended follow up appointments without prompting. *n* = 3/19 (17%) were persuaded to attend as a result of the follow‐up call Follow‐up calls were associated with a change in behaviour in only 3% of patients
Stergiopoulos *et al*. (2017)	RCT	Canada	*n* = 166 *n* = 83 CATCH‐ED ( support coordination) *n* = 83 UC (education session and resource guide)	CATCH‐ED: 42.7 SC 15.7 UC: 47.1 13.5 S	Gender: CATCH‐ED: F: 44 (53%) UC: F: 37 (44.6%)	12 months	CATCH‐ED support co‐ordination interventio	Primary outcome: Frequency of ED visits reduced by 14% (not statistically significant) Secondary outcomes: Overall health outcomes were no different between the two groups
Kahan *et al*. (2016a)	Qualitative Interviews FGs with TCMs‡	Canada	*n* = 47 stakeholders	NS	NS	6–9 months	CATCH‐ED support coordination intervention 4–6 months	Themes Meeting complex health and social needs Assisting/encouraging personal growth Increasing perceived social support Service users: System navigation was seen as an important support coordination role; peer support as a potential means of system navigation; An additional support coordination role identified was that of an intermediary and advocate, facilitating communication within the context of a healthcare system
Kahan *et al*. (2016b)	Qualitative Interviews and FGs	Canada	*n* = 20^§^ frequent ED users *n* = 13 service providers	18+	NS	12 months	CATCH‐ED support coordination intervention	Themes Complex health and social needs Stigma and discrimination in healthcare settings Social isolation Personal growth and desire to contribute
Poremski *et al*. (2016)	Qualitative Interviews and FGs	Canada	*n* = 20 frequent ED users 18+ *n* = 13 service providers	18+	NS	6 months	CATCH‐ED support coordination intervention	Themes Facilitators of care continuity The working relationship Coordinated service navigation Seamless transitions to future service providers Barriers to care continuity Difficulty engaging frequent emergency department users Short service duration Multiple providers and lack of accountability

† Primary care provider.

‡ Transitional case managers.

^§^ In the original study there were *n* = 83 CATCH‐ED frequent emergency department users and *n* = 83 usual care. The *n* = 20 interviews came from first group.

## RESULTS

In this systematic review, eight databases were searched retrieving 9224 records, resulting with 10 included articles (seven quantitative and three qualitative papers) representing seven studies. Although the search strategy included studies from all countries, only studies from USA, Australia and Canada met the inclusion criteria. All studies included an evaluation of a communication pathway to assist with connecting service users with community mental health, outpatients' services and/or a general practitioner (primary care physician). All participants had presented to an ED with mental health concerns or suicidality. All studies after critical appraisal scored higher than 55% with most scoring 80–92%. Characteristics of the seven quantitative and three qualitative papers are displayed in Table 2 and discussed below.

It is important to note that only three studies elicited lived experience perspectives on communication strategies (Grupp‐Phelan *et al*. 2012, 2019; Kahan *et al*. 2016a, 2016b; Poremski *et al*. 2016) and only one study included a researcher with lived experience (Kahan *et al*. 2016b). Two of these studies incorporated the carer/parent/service user voice (Grupp‐Phelan *et al*. 2012, 2019) and one study consisted of service user interviews (Kahan *et al*. 2016b; Poremski *et al*. 2016). The three qualitative papers (in one study) reporting interviews with service users raised the themes of short service duration, social isolation, complex health and social needs and stigma in the healthcare setting (Kahan *et al*. 2016a; Kahan *et al*. 2016b) along with service users reporting personal growth and the desire to give back (Kahan *et al*. 2016a, 2016b).

### Support coordination approach

Three studies (Adams & Nielson 2012; Griswold *et al*. 2010; Kahan *et al*. 2016a, 2016b; Poremski *et al*. 2016; Stergiopoulos *et al*. 2017) reported on a group of interventions which authors categorized as support coordination. Not all studies explicitly used the term support coordination, but these interventions included at least two of the following elements which can be defined as support coordination (phone follow‐up, interviews, arranging appointments, referrals to other services, team meetings and home visits).

Adams and Nielson (2012) used a support coordination process for people who were repeatedly presenting to a community‐based psychiatric ED in the United States after recent hospitalization. Along with support coordination (which included phone calls, making appointments with community mental health services, discussions around suitable follow‐up) staff deferred discharge to ensure support coordination was conducted and appropriate follow‐up care was in place. Authors of this study reported that ED psychiatric repeat presentations for people seeking help decreased from 6.51% in 2010 to 4.3% during the first six months of 2011^3^.

Griswold *et al*. (2010) employed care navigators to assist people presenting for emergency care in a facility in the United States. Care navigators employed support coordination, for example, interviews, phone contact, home visits, and peer connection to mental health sites and social services. Compared with usual care (e.g. psychiatric assessment, therapeutic approaches, linkages to community mental health services, and if requested referral to primary medical care), at one‐year follow up a higher number of participants were linked to primary care with the support coordination intervention group *n* = 63 (62.40%), compared to usual care *n* = 38 (37.6%).

One study comparing support coordination with education was reported in four papers (one paper reporting on results of an RCT; Stergiopoulos *et al*. 2017, and three papers reporting on qualitative findings; Kahan *et al*. 2016a, 2016b; Poremski *et al*. 2016) and was conducted in six hospital EDs in Canada. The study reported on the Coordinated Access to Care from Hospital Emergency Departments (CATCH‐ED) a support coordination intervention compared to an education session and a resource guide, with *n* = 166 participants who were >18 years. The CATCH‐ED intervention incorporated support coordination aspects, including community supports such as primary care, peer support, counselling and other services as needed, over four to six months. The outcome of this RCT was a reduction by 14% of people repeating presentations to ED, however, this was not considered statistically significant. Authors of this study reported that support coordination compared with an education intervention did not reduce ED presentations or improve health outcomes.

### Telephone follow‐up

One study (Hopper *et al*. 2011) conducted in Australia reported on a telephone only follow‐up intervention for adolescents presenting at a paediatric ED. Only *n* = 3/19 young people were prompted to attend a follow‐up appointment as a result of the follow‐up phone call. As stated by Hopper *et al*. (2011), this sample was limited as it was conducted at one site only and the study was subsequently not repeated. Results may have been compromised due to ceiling effects as 83% attended follow‐up appointments without requiring a phone prompt. This is a high rate of appointment attendance (without intervention) and may be due to the age of the person with lived experience (adolescent) and the involvement of carer/parents.

### Motivational interviewing (counselling)

Two papers reported on RCTs with motivational interviewing interventions and SC. Grupp‐Phelan *et al*. (2019) reported on an RCT study/intervention titled *Suicidal Teens Accessing Treatment After an Emergency Department Visit (STAT‐ED)*. This study included some phone follow‐up, along with motivational interviewing as a person‐centred approach to encourage family engagement, problem solving and referral assistance, compared with usual care, for example, brief mental health care consultation and referral, for participants 12–17 years old. Both groups (*n* = 79: STAT‐ED: *n* = 80 UC) had significantly lower rates of depression and suicidal ideation at two and six months. Although over time, the STAT‐ED group had more participants that had attended one or two appointments, the results were not significant.

Grupp‐Phelan *et al*. (2012) also conducted a pilot RCT with paediatric participants (between 12 and 19 years) presenting to the ED in the United States who were screened for suicidal risk factors and/or alcohol use or depression. Participants randomized to the TeenScreen‐ED *n* = 11, an intervention designed to address logistical and attitudinal barriers to engage in follow‐up care, were more likely to schedule an appointment (73%) and attend the appointment (64%) than usual care *n* = 13 (15.4%), which consisted of an interview with an ED psychiatrist and an outpatient referral. At two‐month follow‐up the TeenScreen‐ED group depression levels had improved non‐significantly with the Centre for Epidemiologic Studies–Depression scale (CES‐D scores); depression scores had increased slightly for the usual care group.

### Electronic record enhanced strategy

Esposito *et al*. (2020) reported on strategies to improve communication follow‐up with primary care physicians following presentations at a paediatric ED in the United States. This quality improvement initiative was evaluated through a chart review three months prior and 12 months post the presentation. Participants completed screening for mental health concerns with the Behavioural health screen (BHS‐ED) and then were included in the study. The electronic record enhanced strategy included electronic record alerts, templates for recording contact with primary care physicians and capacity for primary care physicians to access medical records remotely. Communication to primary care physicians increased from 1% at baseline to 40% at post intervention (average 26%). Prior primary care physician follow‐up was 5%, whereas post‐intervention scores were significant at 67%. Repeat presentation rates to the ED within 4 weeks were 4% prior to the study with post‐intervention scores at 0.5%.

## DISCUSSION

This review examines the effectiveness of continuity of care as experienced by people presenting to the ED with mental health concerns at discharge where care is transferred to community mental health care, outpatients or general practitioners. This review has identified strategies such as support coordination, motivational interviewing, electronic record enhancement and phone follow‐up as promoting continuity of care for people presenting at the ED with mental health‐ or suicide‐related distress. Such strategies may also enhance other outcomes such as reduced levels of depression and repeated presentations to the ED.

In the United States, 5–7% of all ED presentations are people in mental distress (Kalb *et al*. 2019), with around 4% in the United Kingdom (Barratt *et al*. 2016). Over the last 5 years, within Australia, this figure is 3.6% (Australian Institute of Health and Welfare 2020a, 2020b; Magarey 2019; Washington 2020), with a majority of people seeking assistance with self‐harm or suicidal crisis (Australian Institute of Health and Welfare 2019a, 2019b; Duggan *et al*. 2020). An important aspect of understanding the effectiveness of people's use of EDs in crisis would be to measure rates of re‐presentation and also uptake of aftercare arrangements. Yet it is common for EDs not to have systems in place to identify trends for people representing. (Boudreaux *et al*. 2011). This is a critical issue, as needing to re‐present to the ED is a clear indication of peoples' needs not being met by the wider support system.

Four studies (Esposito *et al*. 2020; Grupp‐Phelan *et al*. 2019, 2012; Hopper *et al*. 2011) included in this review whose participants were adolescents included mental health screening and varying interventions, for example, electronic record alerts, phone follow‐up, motivational interviewing plus support coordination, whereas studies with adult participants focussed on support coordination only. It is accepted that a young person may present to the ED with a mild medical complaint, but also have a mental health issue (Nager *et al*. 2017). As their mental health literacy is just emerging, they may be unaware that a medical complaint could be masking an underlying mental health issue.

Depression is a leading cause of disability (World Health Organization 2021c), and suicide is the fourth highest cause of death for adolescents between 15–19 (World Health Organization 2021b); this is aligned with the low rates of help‐seeking and disclosure for this age group (Radez *et al*. 2021). Follow‐up interventions assist with communication and, therefore, better aftercare for those presenting to the ED in distress. Motivational interviewing is about readiness for change. A supportive person to speak to with the family will work to identify changes that the person can bring into their life and assist with exploring the person's sense of capacity to change. These follow up strategies were shown to be valuable to improve after care arrangements with three of the included studies in this review (Esposito *et al*. 2020; Grupp‐Phelan *et al*. 2012, 2019).

Promoting person‐centred care and a rights‐based approach with the lived experience voice in the research space is being called for internationally by governments and people with lived experience (Australian Commission on Safety and Quality in Health Care 2011, 2017; World Health Organization 2021a). Yet, as can be seen by the results of this review, the lived experience voice is still overshadowed by that of clinicians (Banfield *et al*. 2018). People with a cognitive impairment, an intellectual disability or a mental health issue are entitled to participate in research and to be supported to play an active and central role in their own healthcare (National Health and Medical Research Council 2018). As recommended in the National Safety and Quality Health Service *Clinical Governance Standards* (2017) to enable person‐centred care, health services must partner with people with lived experience in all aspects of health care involving them in ‘planning, design, delivery, measurement and evaluation of systems and services’ (p. 14). The mental health sector in Australia is undergoing a significant shift in this area with all national and state mental health plans directing that consultation and inclusion with people with lived experience in the design and strategies of mental health care be implemented (Department of Health 2017; Mental Health Commission 2018; New South Wales Ministry of Health 2018; Northern Territory Government 2019; Primary Health Tasmania 2021; Queensland Mental Health Commission 2018; South Australian Mental Health Commission 2017; Department of Health and Human Services 2015). As person‐centred care promotes empowerment and involvement in decision making, as well as high quality transitions of care across systems (Kelly *et al*. 2019), it is vital that service users are included in research projects focused on improving communication pathways and continuity of care (MacDonald *et al*. 2019; Minshall *et al*. 2020).

### Communication pathways and continuity of care

Clear information exchange via communication pathways between all parties involved (patient, clinicians and service providers) enhances and provides the architecture to enable continuity of care. E‐records, like their paper forerunners, are only as helpful as the information entered and the processes implemented. An environment where information sharing is working well, and service provision meets the needs of people requiring care is an ideal environment to enable shared‐care and decision making and therefore shared power (O'Shea *et al*. 2019). The ED, as the portal of crisis health care, requires smooth communication pathways between treating clinicians and towards external service providers to enable seamless continuity of care to occur for the service user. This in turn will increase favourable outcomes for people (Curran *et al*. 2019; Newnham *et al*. 2017) and build a sense of relational trust in the system with power sharing (Wierdsma *et al*. 2009). It is clear from this review that further work and funding needs to be engaged to reach this aspirational goal.

This evidence suggests that lived experience perspectives of service users can highlight areas of concern that need to be addressed to improve mental health outcomes following presentation to an ED. Coupled with the identified desire of service users to contribute to reform (redesign), this represents a powerful opportunity to understand the consequences of different approaches and work towards the development of services that are informed by lived experience. Likewise, research reports widely on the benefits of peer support programs in mental health and drug and alcohol addiction programs (Shalaby & Agyapong 2020), mostly providing one‐to‐one peer support in the community mental health setting or in recovery groups. Yet, only two studies in this review mentioned peer support as a valid resource and peer led services, as an intervention around continuity of care within the ED setting, did not appear in the results (Griswold *et al*. 2010; Stergiopoulos *et al*. 2017).

### New ways of thinking

Community‐based alternatives to EDs such as Safe Haven Café, Crisis Stabilization Centres provide non‐clinical style, high‐engagement support in a calm, ‘lounge room like’ space to help individuals in need of urgent mental health care (Mental Health Commission 2021; Neami National 2021). Most of these settings employ staff in designated peer roles, making available mutual peer support skills in combination with trauma informed care principles thus reducing the need for ambulance and police intervention (Black Dog Institute 2020; Consumers of Mental Health Western Australia 2019). When combined with peer work, trauma informed processes make it easier for people to calm, tell their story of recent events and experiences and consider practical steps including supports from individuals and organizations that comprise of over‐arching aftercare connections and arrangements (Black Dog Institute 2020; Consumers of Mental Health Western Australia 2019). As a specific mental health service, these models are likely to have improved capacity to effectively link and communicate aftercare arrangements with service users and community supports compared to EDs. New South Wales Health, in collaboration with *Towards Zero Suicide*, are establishing twenty new services across NSW which similar in design to the SafeHaven initiative and will be staffed by peers in a non‐clinical support setting (New South Wales Ministry of Health 2020). SafeHaven peer‐led services provide an alternative to presenting to an ED when experiencing mental distress and are often open after office hours (New South Wales Ministry of Health 2022) and have the capacity to connect service users to community mental health services. The new Urgent Mental Health Care Centre established in Adelaide is a prime example of offering an alternative to the ED. This centre was established through codesign methodology and innovation by people with lived experience and provides a combination of peer‐led recovery with clinical support (Urgent Mental Health Care Centre 2021) in a scaled down clinical setting.

### Strengths and limitations

One of the strengths of this review is the inclusion of a lived experienced researcher and a lived experience carer in the design and investigation of the topic. To our knowledge, it is the only systematic review focussing on communication pathways for continuity of care between the ED and community mental health services, outpatients and/or general practitioners. This review followed PRISMA guidelines and developed a comprehensive search strategy developed in collaboration with an academic librarian (see Fig. 1) to ensure a thorough and rigorous search using the PICO framework. Two reviewers screened all titles and abstracts and read the full text of results at the full text screen, and cross checked the data extraction and critical appraisal.

Limitations may include the following. Articles were excluded if it was unclear if participants were being discharged from the ED or it was unclear if they were being admitted as an inpatient. Therefore, it is possible that some relevant studies may have been excluded. Future research should clearly identify a system‐wide pathway of transitions of care between EDs, inpatient settings and community supports with a strong focus on service user and carer experience of these transition pathways. Although the term ‘homeless person’ was used in the search strategy research papers included in this review did not address a variety of diverse populations. This highlights a limitation in existing research in this area.

## CONCLUSION

This review contributes to the body of knowledge around communication pathways from the ED at transfer of care to community mental health services, outpatients, or general practitioners. Our goal in this review was to discover the effectiveness of any evaluated communication pathway strategy to enhance continuity of care from the point of discharge from the ED only. Our focus on improved communication pathways was due to documented concerns that service users often experience gaps in service and communication after presenting to EDs for assistance, or that effective help is not provided to them. This review demonstrates that support coordination, counselling initiatives such as motivational interviewing, education or enhanced electronic records sharing can improve continuity of care for people presenting at the ED with mental health concerns or suicidal crisis, and reduce the need to re‐present. These initiatives may also support positive health outcomes such as reduced levels of depression. Therefore, it can be suggested that combining these strategies may improve continuity of care and service user engagement (along with secondary outcomes). It is clear from the results that further research is needed to identify additional strategies and processes to assist with purposeful communication pathways at transfer of care. The seven studies (reported in 10 papers) incorporated in this review attest to the small amount of published research on this topic.

It is clear that people with lived experience have had limited input in this area of research. People who travel the journey of service use can provide the greatest insights in this context. This review reveals some current sector pathways for continuity of care, and promotes consideration for the provision of alternative options for people in distress other than the ED. The limited evidence of effective continuity of care practices connecting people with community mental health services following an ED presentation calls for fresh thinking that is driven by consumers and carer advisors (Johnston *et al*. 2019).

## RELEVANCE FOR CLINICAL PRACTICE

Mental health nurses along with team members see people at the very point of their distress. This systematic review has brought together evidence on evaluated interventions for communication pathways for continuity of care between the ED and community mental health services, outpatients and/or primary care settings. The findings from this review demonstrate ways that mental health nurses can advance and advocate communication pathway interventions to help improve continuity of care for individuals presenting to the ED with mental health and/or suicide related distress. These findings can be used to improve quality of nursing practice linked to service outcomes within the health care system with the aim to mitigate further distress, returning to the ED, as well as follow up care and support in a timely and personalized manner.

## Funding statement

This systematic review is part of a larger mixed methods project looking at the current communication pathways for continuity of care used by clinicians to assist people with a psychosocial disability who present to EDs within Australia and is funded by MIND Australia.

## Ethics approval statement

This systematic review is part of a PhD project which has been approved by the University of South Australia Human Research Ethics Committee (Protocol 203 626).

## Data Availability

The data that support the findings of this study is available on request

## Endnotes

^1^
For the purposes of this article authors will refer to patients or mental health consumers as service users.

^2^
The research protocol was determined by the research team prior to commencing the systematic review (Prospero: CRD42021233857).

^3^
See data extraction table. This study did not report participant numbers.
